# Evaluating bioactive constituents and toxicological effects of aqueous extract of fermented *Pentaclethra macrophylla* seeds in rats

**Published:** 2020

**Authors:** Eziuche Amadike Ugbogu, Chukwumaobim Daniel Nwoku, Victor Chibueze Ude, Okezie Emmanuel

**Affiliations:** 1 *Department of Biochemistry Abia State University, PMB 2000, Uturu, Abia State, Nigeria*; 2 *Department of Biochemistry, Faculty of Pure and Applied Science, Federal University Wukari, PMB 1020, Wukari, Taraba State, Nigeria*; 3 *Department of Medical Biochemistry, College of Medicine Enugu State University of Science and Technology, PMB 01660, Enugu-Nigeria*

**Keywords:** Pentaclethra macrophylla Bioactive compounds, Lipid profile, Hepatic enzyme, Histopathology

## Abstract

**Objective:**

This study aimed at evaluating the bioactive constituents and the toxicological profile of the aqueous fermented seed extract of *P. macrophylla*.

**Materials and Methods:**

The chemical constituents of fermented *P. macrophylla* were assessed using GC-MS. For acute toxicity study, one-time doses of up to 5000 mg/kg of the extract were orally administered to male and female rats whereas 200, 400 and 800 mg/kg of the *P. macrophylla* extract were orally administered daily for 14 days in sub-acute toxicity investigation. Biochemical, haematological and lipid profiles were assessed following standard methods.

**Results:**

Bioactive compounds such as citronellol and oxirane, tetradecyl- (hexadecylene oxide) were identified in the extract. In acute toxicity test, no death or sign of toxicity was identified. For sub-acute study, ALT decreased significantly (p<0.05) while HDL-C had dose-dependent increases. No effect (p<0.05) on haematological parameters except on platelets was found. No histopathological changes were observed.

**Conclusion:**

Our results demonstrated that the extract of fermented *P. macrophylla* caused no toxic effects in the rats at the tested doses. Therefore, they may be termed safe for consumption and therapeutic uses.

## Introduction

*Pentaclethra macrophylla*, an oil bean, is a perennial leguminous tree native to tropical Africa, employed for traditional alternative medicine (TAM) when treating different forms of ailments. Anti-inflammatory, anti-helminthic, abortifacient and analgesic activities of the leaves, stem bark, seeds and fruit pulp extracts have been reported (Akindahunsi, 2004 ▶). The medicinal properties are most likely a direct consequence of the bioactive agents in the different plant parts (Akindahunsi, 2004 ▶; Fungo et al., 2015 ▶). Several studies on the nutritional and physicochemical properties of *P. macrophylla *have already been performed which demonstrated the presence of proteins, amino acids, carbohydrates, fat, fibre, thiamine, riboflavin and niacin (Onwuliri et al., 2004 ▶; Odoemelam, 2005 ▶; Ogueke et al., 2010 ▶; Osagie-Eweka and Alaiya, 2010 ▶; Eze et al., 2014). However, its bioactive constituents have not been studied. Fermented and unfermented *P. macrophylla *seeds contain essentially similar nutrient levels except for carbohydrates which increased slightly in fermented *P. macrophylla *seeds, suggesting that fermentation has no significant effect on nutrient content (Enujiugha, 2003 ▶; Monago et al., 2004 ▶). The level of vitamins in *P. macrophylla* may be elevated when they are fermented, particularly thiamine, riboflavin and niacin (Achinewhu, 1983 ▶). Due to the high protein content (Achinewhu, 1983 ▶), *P. macrophylla* is considered an important and affordable protein source for communities whose staple foods lack proteins. However, a more recent work documented lowering of* P. macrophylla* protein content during fermentation with a little elevation in carbohydrates and oil concentration (Akindahunsi, 2004 ▶).

In Southern rain forest zone of West Africa, particularly in the Eastern region of Nigeria, about 50 million people depend on different parts of *P. macrophylla *tree for food, and charcoal making. The seed pulp of *P. macrophylla* is consumed boiled or roasted, and more frequently undergo fermentation prior to their consumption. Among the Igbos of Nigeria, its commonest folklore culinary application is the fermented seed product popularly called *Ugba*, which has a meaty taste and is served both as a delicacy and a soup flavoring agent (Mbajunwa et al., 1998 ▶; Enujiugha and Akanbi, 2005 ▶). There have not been any established cases of acute or chronic poisoning due to Ugba consumption over the years. Interestingly, several health benefits have been reported, including lowering of plasma cholesterol (Monago et al., 2004 ▶), and reducing the risks of ulcer (Ugbogu et al., 2017 ▶), cancer and some tobacco-related diseases (Chidozie, 2006 ▶). However, the presence of saponins (Achinewhu, 1983), a growth depressant, caffeoyl-putrescine (Ugbogu et al., 2017 ▶) and a poisonous alkaloid, paucine (Isu and Njoku, 1997 ▶; Chidozie, 2006 ▶; Ugbogu et al., 2017 ▶), which are toxic and with no nutritive value has been reported. It is assumed that fermentation of *P. macrophylla *seeds significantly reduces the content of the poisonous alkaloid such as paucine via leaching and degradation during the harsh thermal processing and fermentation processes (Onwuliri et al., 2004 ▶). Akindahunsi (2004) ▶ reported no changes in tannin levels, and a marked increase in phytates, after extensive processing of *P. macrophylla.*

The proteolytic fermentation, which is not primed with a starter culture, occurs naturally under alkaline conditions mainly through the activity of species of the genus *Bacillus* (Isu and Njoku, 1997 ▶; Ogueke and Aririatu, 2004 ▶; Okwulehie, 2004 ▶; Kabuo et al., 2007 ▶)*. *However, the presence of *Escherichia coli* and *Staphylococcus aureus* (Ogueke and Aririatu, 2004 ▶), *Aspergillus flavus*, *Aspergillus niger*, *Penicillium chrysogenum* and *Fusarium spp* (Azubuine and Isu, 2006 ▶; Ogueke et al., 2010 ▶) as well as *Candida tropicalis* and *Geotrichum candidum* (Ejiofor et al., 1987 ▶; Ogueke et al., 2010 ▶) has been reported. This poses potential health risks to consumers, but the subsequent heat treatment during cooking when *Ugba* is used as soup condiments, may eliminate these microorganisms.

No detailed and definite toxicological study has elucidated the safety of *Ugba* consumption. To lay these assumptions to rest, this paper therefore seeks to explore the possible toxicity of aqueous extracts of fermented *P. macrophylla* by analyzing blood, hepatic and renal profiles in rats. The histomorphological changes in the liver and kidneys were also evaluated to corroborate the biochemical parameters.

## Materials and Methods

**Sampling of** ***P. macrophylla***

*P. macrophylla* seeds were sourced from Eke Okigwe, Okigwe Local Government Area, Imo State, Nigeria. They were carefully sorted to remove contaminants. *P. macrophylla* seeds were authenticated at University of Nigeria Nsukka and deposited in the herbarium (No. UNN/138a). 

**Sample processing**

For seed processing, the method of Isu and Ofuya (2000) ▶ was adopted with some modifications. The seed coats were softened by prolonged boiling (12 hr) and the released embryos were boiled for another 4 hr after fine slicing. Thereafter, the seed particles were washed thoroughly for 4 to 5 times with distilled water to reduce the bitter taste, wrapped in *Alchornea laxiflora *Benth leaves (okpopia leaves), packed in aerated bags. The wrapped sliced seeds were allowed to undergo fermentation at ambient temperatures and used after 3 days.

**Preparation of** ***Ugba*** **seed extract**

Exactly 200 g of the fermentation product (*Ugba*) was placed in 500 ml of distilled H_2_O for extraction at 25°C for 18 hr. Then, the solids were removed by filtration. The filtrate was immediately used for analytical purposes or further experiments.

**Identification of the bioactive compounds of** ***P. macrophylla***

To identify the chemical constituents of the aqueous extract of fermented *P. macrophylla *(*Ugba*), GC-MS analysis (Agilent 7890A-5975C GC-MS system) with column, HP5 (30 m x 0.25 mm x 0.25 µm); and carrier gas, ultra-pure helium at a flow rate of 1.0 ml/min and a linear velocity of 37 cm/s was used (Ugbogu et al., 2019 ▶). The temperature of the injector was 250^o^C; oven temperature, was initially 110^o^C and increased at 10 ^o^C/min until 280^o^C (7 min at each increment). The injection volume was 0.5 µl in the splitless mode with a split ratio of 10:1; mass spectrometer and 70 eV electron ionization mode. The retention times as well as mass spectral data and fragmentation pattern were directly compared with those available in the National Institute of Standards and Technology (NIST) database for identification of the chemical constituents (Ugbogu et al., 2019 ▶).

**Animal procurement and housing**

The study was performed in rats weighing from 140 to 180 g. The animals were sourced from the University of Nigeria. Animals were acclimatized for a period of two weeks and fed on grower mash and allowed free access to water in rooms that were well ventilated at ambient temperature with 12/12 hr light/dark condition (Hajihosseini and Setorki, 2017 ▶; Ugbogu et al., 2018 ▶; Ugbogu et al., 2019 ▶). The animal studies were performed according to the United States National Institutes of Health Guidelines for Care and Use of Laboratory Animals in Biomedical Research (NIH, 1985 ▶).

**Acute toxicity study**

Assessment of acute toxicity of *Ugba* aqueous extract was performed following the guideline described by Organization for Economic Cooperation and Development (OECD) (OECD, 2001) guideline 423 with minor modifications. The rats were randomly divided into six groups of 12; each group consisted of 6 male and 6 female rats. Post overnight fasting, one-time doses of 500, 1,000, 2,000, and 5,000 mg/kg of *Ugba* extracts were orally administered (gavage) while the control group received 0.25 ml/kg of distilled water. Behavioral changes and mortality of the animals were observed for 24 hr and thereafter, for a period of 14 days. 

**Sub-acute toxicity study**

Twenty-four rats were randomly placed into four groups of six each. Group I served as a control which did not receive aqueous extract of *Ugba*. Group II, III and IV were administered with 200, 400 and 800 mg/kg *Ugba* extract, respectively. Extract administration was performed daily by oral gavage over a period of 14 days. The time and doses used in this study were selected based on previous studies (Traesel et al., 2016 ▶; Ugbogu et al., 2018 ▶; Ugbogu et al., 2019 ▶) and the results of the present acute toxicity study. Thereafter, the body weight of the rats was determined. After overnight fasting, they were anesthetized and sacrificed. The spleen, heart, lungs, kidney and liver were excised, cleaned by blotting with filter paper and weighed to determine their relative weight. For histological examinations, the organs were fixed in 10% formalin and embedded in paraffin wax. Whole blood was collected by cardiac puncture using sterile needles and kept in sterile plain and EDTA bottles for analyses. 

**Determination of biochemical, haematological and histopathological parameters**

The enzymatic activities of the liver transaminases (aspartate aminotransferase (AST), alanine aminotransferase (ALT) and alkaline phosphatase (ALP)) as well as triacylglycerol (TAG), total cholesterol (TC), low-density lipoprotein cholesterol (LDL-C) and high-density lipoprotein cholesterol (HDL-C) were determined spectrophotometrically using the kits from Randox Laboratory Ltd., Co. Antrim (United Kingdom) according to the manufacturer’s instruction. Urea, creatinine, Na^+^, K^+^, Cl^-^ and HCO_3_^-^ were spectrophotometrically determined using Teco test kits (Teco Diagnostics, USA). Haematological parameters were determined according to the method previously described by Afia and Momoh (2006) ▶ using the BC-3200 Auto-Haematology Analyzer. To study the histopathology of the liver and kidney, 5-μm thick sections were stained with haematoxylin and eosin. The sections were viewed by an experienced pathologist using optical microscope (Fisher, 2002).

**Statistical analysis**

Analysis of data was performed by one-way analysis of variance (ANOVA) with Tukey *post-hoc* test. A p-value of ≤0.05 was considered statistically significant. 

## Results

GC-MS analysis of an aqueous fermented seed extract of *P. macrophylla *revealed the presence of nine chemical compounds (Table 1).

**Figure 1:**
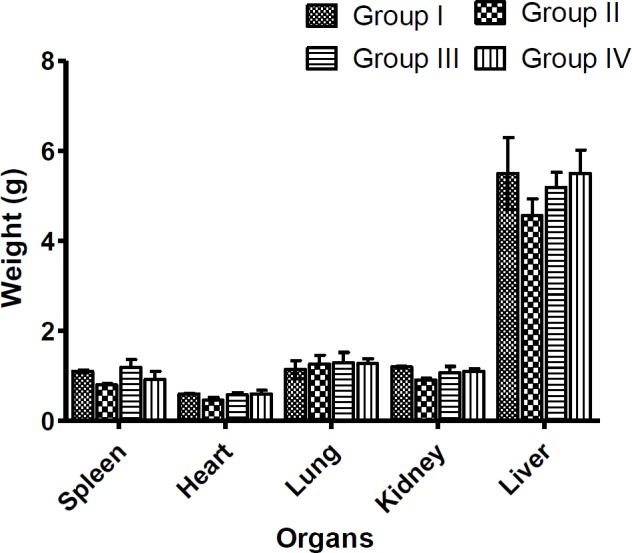
Effect of an aqueous *Ugba* extract on the relative organ weight. Rats were administered with 0.25 ml/kg of distilled water (Group I (Control)), aqueous fermented seed extract of *P. macrophylla* for 14 days at doses of 200 (Group II), 400 (Group III) and 800 (Group IV) mg/kg BW. The rats were sacrificed, and the organs were carefully removed and weighed. Values represent the mean±SD for n=6

Table 2 summarizes the result of the acute toxicity study of the *Ugba*extract in rats. Neither behavioural changes nor mortality was observed in the rats even at the highest dose of the *Ugba*extract (5000 mg/kg body weight). Furthermore, no significant change (p<0.05) in the relative weight of the spleen, heart, lung, kidney and liver even at the highest dose (800 mg/kg body weight) was observed when compared to the control group (Figure 1).

**Table 1:** GC-MS spectral analysis of *Ugba* extract

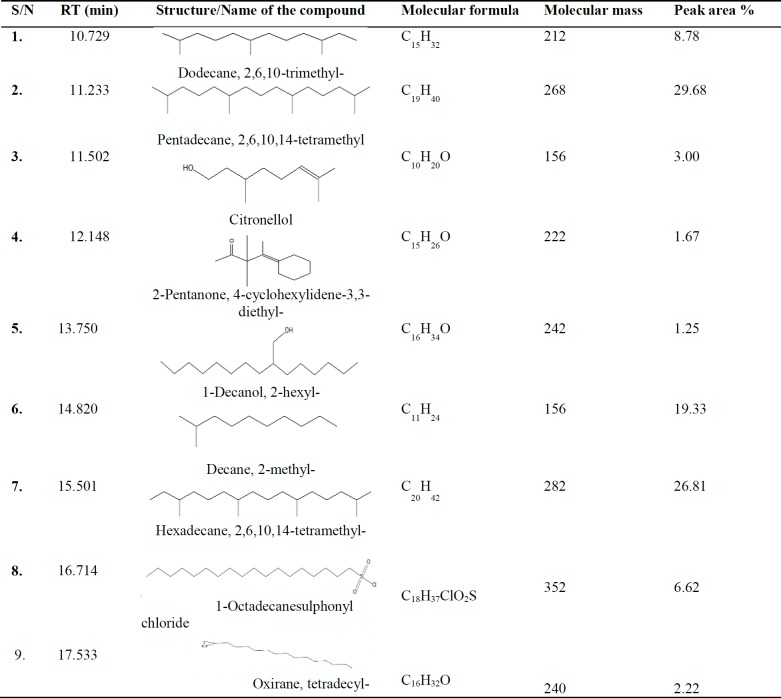

**Figure 2:**
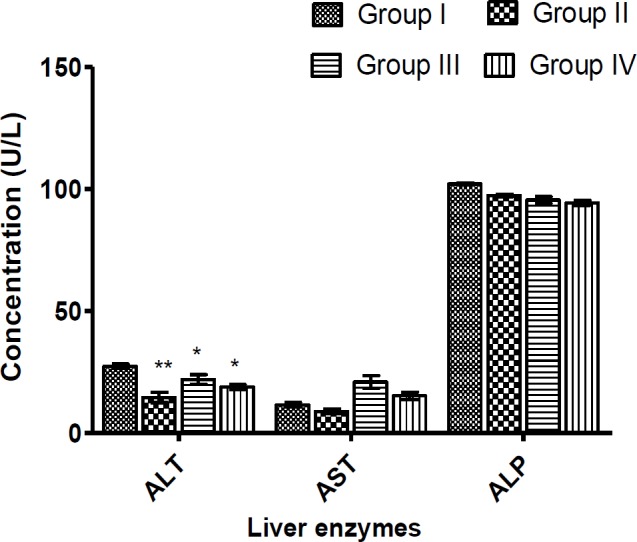
Effects of *Ugba* extract on hepatic enzymes. Rats were administered with 0.25 ml/kg of distilled water (Group I (Control)), aqueous fermented seed extract of *P. macrophylla* for 14 days at doses of 200 (Group II), 400 (Group III) and 800 (Group IV) mg/kg BW. The rats were sacrificed, and the hepatic enzymes were determined in the blood serum. Values represent the mean±SD for n=6. Asterisk (*) indicates significant difference at p<0.05 and ** indicates significance at p<0.01 difference compared to control

Daily administration of an *Ugba* extract to rats, resulted in significant reductions (p<0.05) in the ALT compared to the control group (Figure 2). ALP activity decreased in a dose-dependent manner, but the level of the decrease in the activity was not statistically significant (p>0.05) (Figure 2).

**Figure 3:**
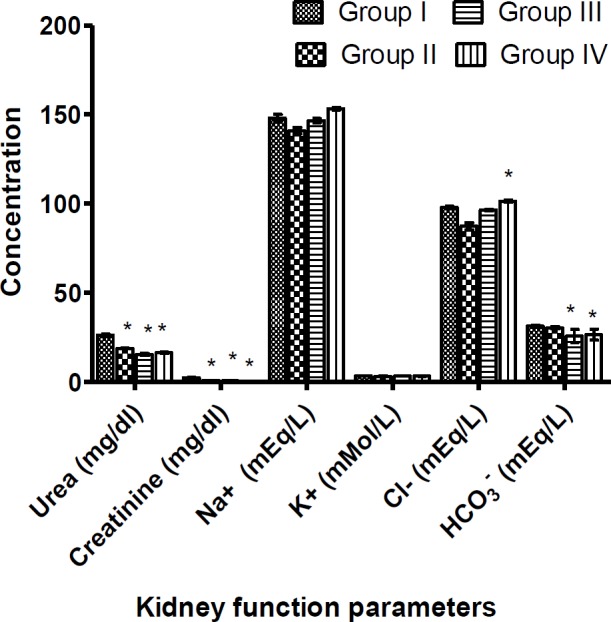
Effects of *Ugba* extracts on kidney function parameters. Rats were administered with 0.25 ml/kg of distilled water (Group I (Control)), aqueous fermented seed extract of *P. macrophylla* for 14 days at doses of 200 (Group II), 400 (Group III) and 800 (Group IV) mg/kg BW. The rats were sacrificed, and the kidney function parameters were determined in the blood serum. Values represent the mean±SD for n=6. Asterisk (*) indicates significant difference at p<0.05 compared to control

Furthermore, creatinine and urea concentrations decreased significantly in all test groups (p<0.05) (Figure 3). In addition, no significant variations (p<0.05) in the serum levels of serum electrolytes (Na^+^, and K^+^) were observed (Figure 3). However, a significant decrease (p<0.05) in the serum level of HCO_3_^-^ was observed when compared to the control group (Figure 3). 

**Figure 4:**
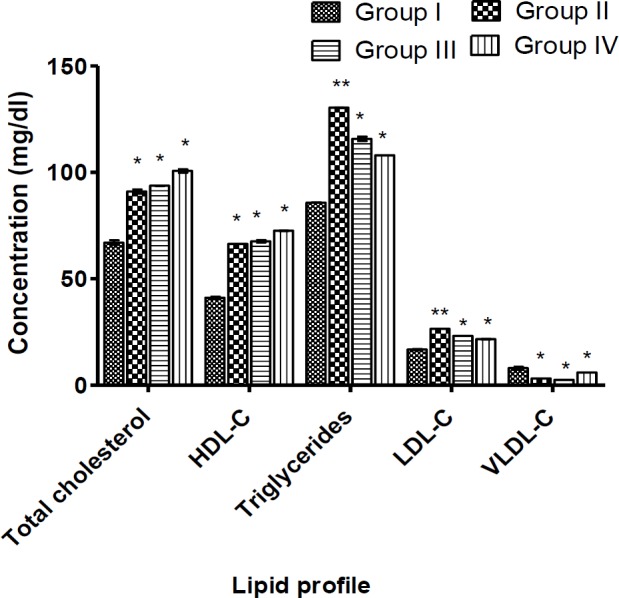
Effect of *Ugba* extract on the lipid profile

Serum total cholesterol (TC), high-density lipoprotein (HDL), triglyceride and low-density lipoprotein (LDL) significantly increased (p<0.05) after 14 day oral administration of the extract in a dose-dependent manner (Figure 4). The serum level of very low-density lipoprotein (VLDL) decreased significantly (p<0.05) when compared to the control group.

Daily administration of *Ugba* extract to rats resulted in a non-statistically significant (p>0.05) reduction in PCV, Hb and RBC (Table 4).

Increases in the level of platelets, lymphocytes and eosinophils were observed whereas a statistically non-significant (p>0.05) decrease in the levels of total WBC and neutrophils was found. 

**Figure 5:**
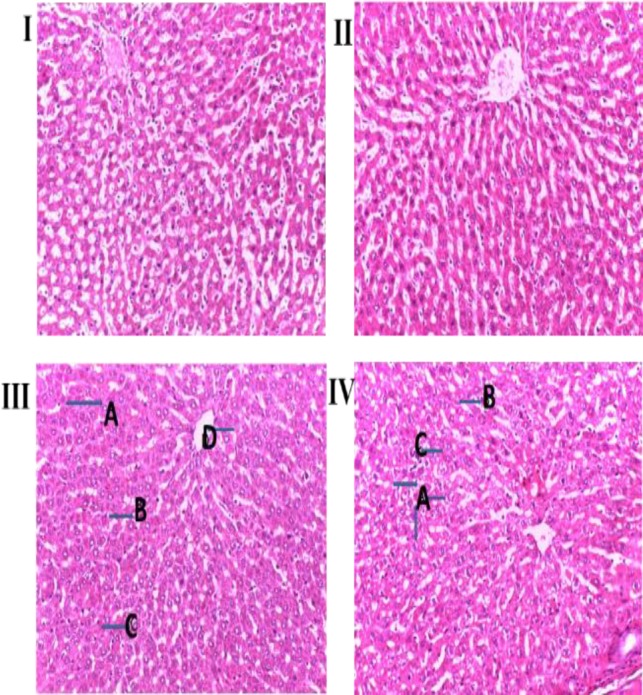
Micrographs of the liver sections obtained from untreated (control) and *Ugba* extract-treated Wistar rats. Rats were administered with 0.25 ml/kg of distilled water (Group I (Control)), aqueous fermented seed extract of P. macrophylla for 14 days at doses of 200 (Group II), 400 (Group III) and 800 (Group IV) mg/kg BW. The rats were sacrificed, and the liver sections were stained with haematoxylin and eosin staining (H&E) solution and observed under an Olympus light microscope equipped with a camera, at a magnification (40×). Alphabets A to D indicate points that appeared to have slightly dispersed histoarchitecture in the liver micrograph

**Figure 6:**
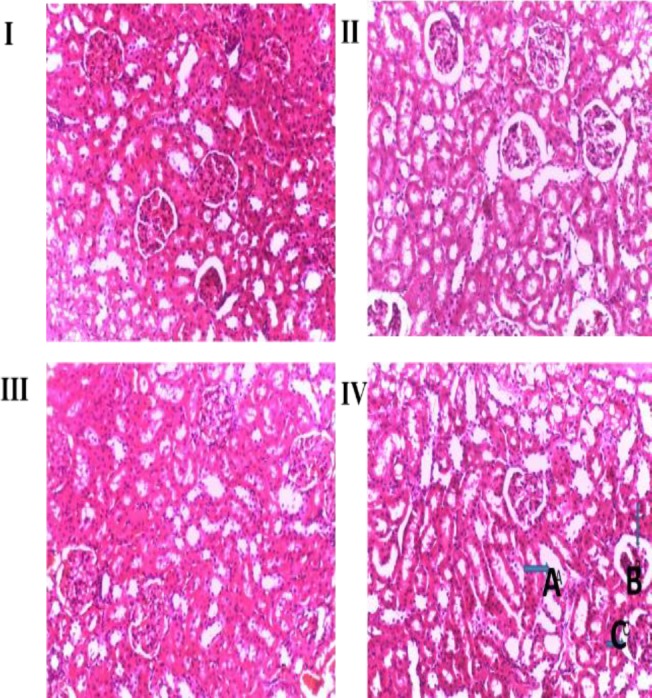
Micrographs of the kidney sections obtained from untreated (control) and *Ugba* extract-treated Wistar rats. Rats were administered with 0.25 ml/kg of distilled water (Group I (Control)), aqueous fermented seed extract of P. macrophylla for 14 days at doses of 200 (Group II), 400 (Group III) and 800 (Group IV) mg/kg BW. The rats were sacrificed, and the kidney sections were stained with haematoxylin and eosin staining (H&E) solution and observed under an Olympus light microscope fixed with camera, at a magnification (40×). Alphabets A to C indicate points that appeared to have slightly dispersed histoarchitecture in the kidney micrograph

The effect of *Ugba* extract administration on the histopathological parameters of the liver and kidney of rats are given in Figures 5 and 6. The micrographs of the liver (Figure 5) and kidney (Figure 6) did not reveal any hepatotoxic or nephrotoxic effects after the 14 day period

**Table 2:** Acute toxicity results of *Ugba* extract

**Group**	**Dose (mg/kg)**	**D/T**	**Signs of toxicity**
**I**	0.25 ml (H_2_O)	0/12	No observable toxic sign
**II**	500	0/12	No observable toxic sign
**III**	1000	0/12	No observable toxic sign
**IV**	2000	0/12	No observable toxic sign
**V**	5000	0/12	No observable toxic sign

Rats were administered aqueous fermented seed extract of *P. macrophylla* for 14 days at doses of 500, 1000 and 2000 and 5000 mg/kg BW and assessed for toxicological symptoms. D/T: Number of rat deaths/Total number of rats included in this group.

Rats were administered with 0.25 ml/kg of distilled water (Group I (Control)), aqueous fermented seed extract of *P. macrophylla* for 14 days at doses of 200 (Group II), 400 (Group III) and 800 (Group IV) mg/kg BW. The rats were sacrificed, and the lipid profile assessed in the blood serum. Values represent the mean±SD for n=6. Asterisk (*) indicates significant difference at p<0.05 and ** indicates significance at p<0.01 compared to control.

**Table 3:** The effect of *Ugba* extract on body weight of rats after 14 days of administration

**Parameter**	**Group I (Control)**	**Group II** **(200 mg/kg)**	**Group III** **(400 mg/kg)**	**Group IV** **(800 mg/kg)**
Weight at day 1	156.00±4.54	161.67±3.31	160.33±4.50	144.40±1.53
Weight at day 14	180.20±13.58	178.33±7.64	178.60±2.34	171.67±3.71
Weight gain (g)	24.20	16.66	18.33	27.27

Rats were weighed prior to the initial administration of aqueous fermented seed extract of *P. macrophylla* at doses of 200, 400 and 800 mg/kg BW for 14 days and weighed again before scarification. Values represent the mean ± SD for n=6.

**Table 4:** Effects of *Ugba* extract on haematological parameters

**Parameter**	**Group I (control)**	**Group II (200 mg/kg)**	**Group III (400mg/kg)**	**Group IV (800 mg/kg)**
PCV (%)	50.00±2.00	45.03±1.50	46.57±1.16	48.47±1.17
Hb (g/dl)	14.50±1.10	12.40±0.44	13.03±0.15	14.03±1.76
RBC (x10^12^/l)	8.70±0.20	7.55±0.40	7.22±0.18	7.68±0.48
MCV (fl)	60.00±0.30	58.50±1.42	66.53±1.19	66.90±1.25
MCH (pg)	16.50±0.50	16.16±0.37	18.28±0.15	17.80±0.43
MCHC (g/l)	278.00±5.00	275.33±5.80	273.33±2.52	273.33±8.62
WBC (x10^9^/l)	17.50±2.30	14.15±0.18	17.40±1.11	14.83±0.47
Lymphocyte (%)	43.30±2.70	48.67±1.15	46.00±3.00	51.33±1.53
Neutrophil (%)	49.00±2.10	47.00±3.61	46.00±1.00	41.33±1.53
Monocyte (%)	5.50±1.00	4.33±2.08	4.33±1.53	4.67±0.58
Eosinophil (%)	1.60±0.10	1.67±1.15	2.67±2.08	3.00±1.00
Basophil (%)	0.00±0.00	0.00±0.00	0.00±0.00	0.00±0.00
Platelet (x10^9^/L)	560.00±40.23	660.33±64.94*	592.33±113.58	686.33±36.50*

Rats were administered with aqueous fermented seed extract of *P. macrophylla* for 14 days at doses of 200, 400 and 800 mg/kg BW. The rats were sacrificed, and the haematological parameters were assessed. Values represent the mean±SD for n=6. Significant differences compared to control at p<0.05, are indicated with asterisk (*).

## Discussion

*Ugba, *a traditional snack with a meaty taste is used for delicacy and soup flavoring in mostly Southern Nigeria (Mbajunwa et al., 1998 ▶; Enujiugha and Akanbi, 2005 ▶). Due to the non-uniform and non-standardized procedures (Njoku and Okemadu, 1989 ▶), the production process as well as the microbial population (NIH, 1985 ▶; OECD 2001 ▶; Afia and Momoh, 2006 ▶) involved in the fermentation process raises questions on product safety, quality and inconsistency. The present study was performed to increase our knowledge about the safety of *Ugba* for human consumption.

Two of the nine chemical compounds identified in *Ugba*, were reported to have pharmacological activities. Citronellol possesses anti-cancer, anti-inflammatory, anti-oxidant and wound healing potential (Zhuang et al., 2009 ▶) and oxirane, and tetredecyl- have been shown to possess anti-microbial properties in a previous study (Manilal et al., 2011 ▶).

Acute toxicological assessment is often employed as a pilot test when investigating the toxicity of substances and aims at deciphering the threshold of tolerance in an animal model (Ashafa et al., 2012 ▶). Ugba induced neither mortality nor behavioral changes or signs of malaise even when rats were fed with 5000 mg/kg body weight. Adverse effects such as restlessness and anorexia were not observed even at 5000 mg/kg. Following the guidance document for testing acute oral toxicity by Organization for Economic Corporation and Development (OECD, 2001 ▶), compounds with LD_50 _values of <2g/kg BW are generally considered relatively safe and doses >5g/kg BW are not considered dose-related. From the available indications, *Ugba* extract is tolerated well up to about 5 g/kg BW and therefore, dosages up to 800 mg/kg BW were chosen for a sub-acute toxicity study.

Organ weight provides a sensitive measure of toxicological assessment (Ugbogu et al., 2016 ▶). After a 14 day period of *Ugba* treatment, weight of body of the rats and that of the organs exhibited a normal incremental pattern without marked differences between treated and control groups (Table 3 and Figure 1). However, a critical analysis showed that the increase in body weight over time was dose-dependent, whereby the 800 mg/kg rats group gained the highest weight (27.27 g). Thus, treatment with *Ugba* extract did not result in a loss of appetite. Another plausible inference is the lipogenic potential of the *Ugba* which agrees with the observations from the lipid profile assessments in the current study (Figure 4). Although the mechanism is not confirmed, the hyperlipidemic effects of *Ugba* may be due to the high content of fatty acid of *P. macrophylla* which already was reported (Jones et al., 1987 ▶; Udosen and Ifon, 1990 ▶). Thus, there are strong indications that these extracts portend risks of hypercholesteroleamia at high doses, increasing the risks of atherosclerosis.

Assay of biochemical parameters was conducted to evaluate the health of the liver and the kidneys (Figure 2 and 3). The presence of normally tissue-specific enzymes and/or metabolites in the extracellular fluid provides clinical diagnostic insights into tissue injury (usually cell membrane disruption) and pathology. Hitherto compartmentalized enzymes exude from the tissue into the blood leading to increased serum level (Akanji et al., 2008 ▶). The liver which is responsible for energy metabolism and system detoxification is delicate. The liver function tests (ALT, AST and ALP assays) revealed a toxin-triggered siege on the liver’s integrity, albeit normal functioning of the liver. ALT and ALP displayed decreasing trends suggesting a hepatoprotective potential for the extract. Although significant dose-dependent increases in serum AST levels in extract-treated animals indicated hepatic impairment, an increasing ALT level is considered a relevant endpoint to determine hepatic damage (Ozer et al., 2008 ▶). Since plasma AST and ALT concentrations increase in the events of inflammation of both cardiac and hepatic tissues (Wasan et al., 2001 ▶; Witthawasku et al., 2003 ▶), myocardial infarction is not very likely to occur. The renal profile parameters such as urea, creatinine, Na^+^, K^+^, Cl^-^ and HCO_3_^-^ demonstrated reduced values in serum after administration of the aqueous *Ugba* extract for 2 weeks. All the reductions were significant, except for Na^+^, K^+^ and Cl^-^. Changes in blood urea, HCO_3_^-^ and creatinine may be used as indices of glomerular filtration rate (GFR). Therefore, the observed decrease suggests improved kidney function (Ravanbakhsh et al., 2016 ▶). This suggests that *P. macrophylla* fermented seed extract may possess reno-protective properties.

This study did not demonstrate notable alterations in investigated haematological indices (Table 4). Usually, haematotoxicity results from changes beyond the reference ranges of these parameters (Dioka et al., 2002 ▶). The alternating reductions and elevations in the measured blood parameters could imply selective systemic toxicity (Ashafa et al., 2012 ▶), perhaps occasioned by variations in cell surface receptors or ion-gated channels. The total RBC count, PCV and Hb levels of extract-treated rats, compared to controls, as well as the MCV, MCH and MCHC (which are individual RBC indices used in anemia classification), were marginally altered, thus validating the fact that the extract does not affect erythropoiesis but protects erythrocytes from oxidative damage (Tarkang et al., 2012 ▶). White blood cells are known to rise in response to toxic environments (Agbor et al., 2005 ▶). At face value, the significant depletion of WBCs generally suggests immunotoxicity, but the equally significant boost in lymphocyte count, the main effector cells of the immune system (Odeghe et al., 2013 ▶), implies the apparent mounting of an immune response by the extract (WHO, 2003 ▶). Neutrophils and monocytes perform phagocytic functions against foreign invaders (Odeghe et al., 2013 ▶, WHO, 2003 ▶); their reduced number in circulation, though insignificant, lend credence to the fact that the fermentative microorganisms found in *Ugba* are non-virulent, and may even be part of the body’s microbiota (Isu and Njoku, 1997 ▶; Ogueke and Aririatu, 2004 ▶; Okwulehie, 2004 ▶; Kabuo et al., 2007 ▶). Eosinophils and basophils are allergy-inducible lymphocytes (Stone et al., 2010 ▶, He et al., 2013), so the unchanging status of basophils and elevation in eosinophil numbers are proof that the extracts contain negligible immunogenic allergens. Platelets protect blood vessels from endothelial damage and mediate blood clotting to repair damaged vessels. Very significant increases in platelet numbers further justify the use of the ripe fruits in wound healing (Akindahunsi, 2004 ▶). It has been posited that hematological alterations in animal studies may be employed in the prediction for translation to human toxicity (Olson et al., 2000 ▶; Adebayo et al., 2005 ▶). Thus, it can be inferred that the extracts possess antioxidant, immuno-modulatory and vaso-protective characteristics.

The outcomes of the haematological and biochemical assays were further confirmed by the homeostatic organs (kidney and liver) histopathological examination. The liver (Figure 5) and kidney (Figure 6) micrograph did not reveal any hepatotoxic or nephrotoxic effects 14 days post-administration of the extracts. A fatty liver is a common toxicant-induced complication; however, the hepatocytes can reverse the associated injury (Treinen-Moslen, 2001 ▶). Interestingly, despite the fattening property of the extract (as noted earlier), the liver did not show any fatty changes or vacuolations. However, the liver was characterized by a slightly dispersed histoarchitecture. The nephrons were visibly intact, with increased extract dosage widening the Bowman’s capsule/glomerular diameter and tubular lumens, all positive factors for kidney function. These observations were confirmed by the relatively stable hepatic and renal profile parameters. 

Given the results obtained in the present study, it can be holistically deduced that the aqueous extract of fermented *P. macrophylla *did not cause any apparent *in vivo* toxicity in the rats at the tested doses. Therefore, they are not likely to have severe toxicological risks for humans. Because of its likely hypercholesterolaemic effects, balanced consumption of *Ugba* should be encouraged pending further research. Moreover, a comprehensive chronic toxicity of fermented *Ugba* extract is essential.
